# In Vivo Studies on the Interaction Between Orally Administered Nitrite and Omeprazole: Beyond Proton-Catalyzed S-Nitrosation

**DOI:** 10.3390/antiox14111307

**Published:** 2025-10-30

**Authors:** Macario A. Rebelo, Alessandra Cássia-Barros, Sandra O. Conde-Tella, Sabrina F. Frugeri, Paula P. Ovidio, Alceu A. Jordão Junior, Cezar Kayzuka, Riccardo Lacchini, Alessandra O. Silva, Carlos R. Tirapelli, Martin Feelisch, Jose E. Tanus-Santos

**Affiliations:** 1 Department of Translational Medicine, Faculty of Medical Sciences, State University of Campinas, Campinas 13083-888, SP, Brazil; m219279@dac.unicamp.br (M.A.R.); a272190@dac.unicamp.br (A.C.-B.); conde@fmrp.usp.br (S.O.C.-T.); 2 Department of Pharmacology, Ribeirao Preto Medical School, University of Sao Paulo, Ribeirao Preto 14049-900, SP, Brazil; sabrinaffrugeri@usp.br (S.F.F.); cezar.filho@usp.br (C.K.); rlacchini@eerp.usp.br (R.L.); 3 Department of Health Sciences, Ribeirao Preto Medical School, University of Sao Paulo, Ribeirao Preto 14049-900, SP, Brazil; ppayao@usp.br (P.P.O.); alceu@fmrp.usp.br (A.A.J.J.); 4 Department of Psychiatric Nursing and Human Sciences, Ribeirão Preto College of Nursing, University of São Paulo, Ribeirão Preto 14049-900, SP, Brazil; 5 Department of BioMolecular Sciences, Faculty of Pharmaceutical Sciences of Ribeirão Preto, University of São Paulo, Ribeirão Preto 14049-900, SP, Brazil; alessandra.osilva@usp.br (A.O.S.); crtirapelli@usp.br (C.R.T.); 6 Clinical & Experimental Sciences, Faculty of Medicine, University of Southampton, Southampton S016 6YD, UK; m.feelisch@soton.ac.uk; 7 Southampton NIHR Biomedical Research Centre, University Hospital Southampton NHS Foundation Trust, Southampton S016 6YD, UK

**Keywords:** nitrite, nitrate, S-nitrosothiols, antioxidant capacity, oxidative stress

## Abstract

Inorganic nitrite contributes to the nitrosation of biomolecules and exerts antioxidant effects. The proton pump inhibitor omeprazole has pro-oxidant effects, inhibits the formation of nitroso species in the stomach, and abrogates the blood pressure-lowering effects of orally administered nitrite. Here, we examine whether a two-week treatment with nitrite leads to tissue nitrosation that scales with local thiol concentrations and whether oral nitrite treatment can prevent the pro-oxidant effects of omeprazole. Male Sprague–Dawley rats received daily doses of omeprazole 10 mg/kg i.p. (or vehicle) and sodium nitrite 15 mg/kg by gavage (or water) for 14 days. Animals were euthanized 6 h after the last nitrite dose, and blood and tissues (brain, heart, and liver) were collected for biochemical analyses. We found that nitrite treatment increased liver nitrite and total nitroso species (RxNO) concentrations approximately eight-fold (with minor increases in other organs), and omeprazole treatment attenuated these effects. Nitrite treatment selectively elevated non-protein thiol concentrations in the liver, but not in animals also receiving omeprazole. Tissue thiol elevation was associated with increased nitrosation of hepatic proteins, which was prevented by omeprazole. Nitrite upregulated mRNA expression of microsomal glutathione S-transferase-1 (Mgst1) and decreased superoxide and hydrogen peroxide production, especially in rats co-treated with omeprazole. While omeprazole increased liver xanthine oxidoreductase (XOR), nitrite treatment attenuated this effect. These results demonstrate that oral nitrite treatment robustly elevates nitrite and RxNO concentrations in the liver, and these effects are associated with increased hepatic glutathione production and an upregulation of Mgst1 expression, counteracting the pro-oxidant effects induced by omeprazole.

## 1. Introduction

The anions nitrite (NO_2_^−^) and nitrate (NO_3_^−^) are the most abundant metabolites of nitric oxide (NO) in mammals and have been explored extensively as potential alternative sources of NO [1,2,3] under pathological conditions where endogenous NO production and/or bioavailability are compromised [4,5]. Nitrite, in particular, may offer some advantages over nitrate because it is more readily bioactivated to NO and is now being considered an important NO storage pool [6,7]. However, nitrite may not only generate NO but also exert antioxidant effects that can counteract pathophysiological mechanisms and contribute to improve disease conditions [8,9,10,11]. Variations in endogenous NO formation rates, along with differences in cells/tissue to take up/handle its oxidation products (nitrite and nitrate), are known to translate into distinct tissue concentrations of these anions across the organ system [12,13,14,15,16]. While recent studies have described heterogenous concentrations of NO metabolites across different organs after oral nitrite administration [17], with major accumulation of nitrite and nitrosylated species in the liver, heart, and muscle [18], it remains unclear whether or not these differences are linked to variations in tissue thiol concentrations, particularly reduced glutathione (GSH), a major defense against free radical injury [19,20,21].

The increase in tissue NO metabolite concentrations observed following oral administration of nitrite differs substantially from that of other administration routes. This is due to the fact that, upon reaching the normally acidic environment of the stomach, nitrite is rapidly protonated to form nitrous acid, which ends up forming NO gas and a series of other nitroxides which, in turn, increase the formation of S-nitrosothiols and other nitrosated species [22]. These chemical transformations and the functional responses to oral nitrite have been shown to critically depend on gastric pH [23,24,25]. Treatment with omeprazole, a proton pump inhibitor that increases gastric pH, attenuates the increase in S-nitrosothiols and the hypotensive response to oral nitrite [24,25,26]. In this respect, we previously reported that a short treatment with omeprazole attenuated tissue accumulation of nitrosylated species in some tissues [22], and this effect could be attributed to impaired gastric formation of S-nitrosothiols and an attenuated reaction of distant tissue targets by transnitrosation reactions. However, it is not known whether omeprazole changes the redox conditions of the tissues, thus affecting the accumulation of NO metabolites. Indeed, omeprazole and other proton pump inhibitors have been reported to have antioxidant [27,28,29] as well as pro-oxidant [30,31,32,33,34] effects. Importantly, while treatment with omeprazole resulted in pro-oxidant alterations with functional consequences [32,33,34] that may contribute to disease conditions [35,36], it is unknown whether treatment with nitrite could prevent pro-oxidant alterations caused by omeprazole.

In the present study, we examine the hypothesis that a two-week treatment with oral nitrite would lead to the accumulation of NO metabolites across different tissues that reflect differences in tissue thiol concentrations and redox conditions. Moreover, we also examine whether omeprazole treatment results in pro-oxidant alterations that could be prevented/reversed by oral nitrite, a compound widely acknowledged to exert antioxidant effects.

## 2. Materials and Methods

### 2.1. Animals

Male Sprague–Dawley rats (250–300 g) were purchased from the breeding colony at the University of São Paulo. They were maintained in a 12 h light/dark cycle at controlled temperature (22–25 °C). The animals had free access to water and standard rodent chow (Nuvilab, Quimtia). All experimental procedures were carried out in agreement with the principles published in the National Institutes of Health Guide for the Care and Use of Laboratory Animals. The study was approved by the Ribeirão Preto Medical School (University of Sao Paulo) animal review ethical committee (on 15 March 2022; protocol number 1032/2021R1).

### 2.2. Experimental Procedures and Materials

This study was designed to evaluate the concentration of NO metabolites in plasma and tissue after 14 days of treatment with sodium nitrite in the presence and absence of co-treatment with omeprazol. This treatment duration was chosen to examine more chronic effects of these drugs, in particular to better reflect the real-life situations under which omeprazole is being used. Sprague–Dawley rats were divided into four distinct groups (n = 10 per group), with a total of 40 animals. This number of animals per group proved to be sufficient to detect statistically significant biochemical differences when they existed in our previous studies. The number of data points for each experiment was not the same due to occasional technical issues or limitations in tissue availability for performing the proposed biochemical measurements. The experimental unit was the animal, and we did not use a computer-generated random sequence to allocate the animals to each study group. However, we found no significant differences between groups in their baseline characteristics. The researcher who performed the biochemical analyses was blinded to the experimental groups.

The four groups of rats were (1) the control, which received water by gavage and vehicle (1 mL/kg PBS, 1 mM, pH 7.4) intraperitoneally for 14 days; (2) sodium nitrite-treated (15 mg/kg by gavage) with vehicle i.p.; (3) omeprazole-treated (10 mg/kg i.p.) with water by gavage; and (4) co-treated with sodium nitrite (15 mg/kg by gavage) and omeprazole (10 mg/kg i.p.), all for 14 days. The animals were euthanized at the end of the treatment period precisely 6 h after the last nitrite dosing [18]. All reagents were purchased from Sigma Chemical Co., (St. Louis, MO, USA), and solutions were prepared immediately before use.

### 2.3. Measurement of Gastric pH

To assess the effect of omeprazole, gastric pH measurement was performed. The abdominal cavity was opened, and the pyloric portion of the stomach was properly secured. Subsequently, an incision was made in the lower portion of the esophagus, and a pH electrode coupled to a previously calibrated bench pH meter (Jenway, Model 3510, Cole Palmer, Bunker, CT, USA) was inserted directly into the gastric lumen [37].

### 2.4. Tissue Harvesting

The rats were anesthetized with ketamine and xylazine (100 mg/kg and 10 mg/kg, respectively), and blood and tissue samples (brain, heart, and liver) were collected 6 h after nitrite was administered for the last time. Blood was collected into heparin tubes via the abdominal vein and centrifuged for 5 min at 1000× *g* at 4 °C. Plasma was separated and aliquoted into amber tubes with 10 mM N-ethylmaleimide (NEM) and 2 mM diethylenetriaminepentaacetic acid (DTPA), which are required for the preservation of nitrosylated species [38]. After blood collection, a catheter was inserted into the aorta, and the rats were perfused with a PBS pH 7.4 solution containing NEM (10 mM) and DTPA (2 mM) to prevent the destruction of nitrosylated species [12]. This procedure was used to ensure that residual blood in harvested organs would be kept to a minimum. At the end of the antegrade perfusion, we harvested the organs, including the brain, heart, and liver, which were frozen in liquid nitrogen and stored at −70 °C for biochemical analysis.

### 2.5. Measurement of Nitrite, Nitrosylated Species (RxNO), and S-Nitrosothiol (RSNO) Concentrations

Concentrations of tissue homogenates or plasma aliquots were analyzed in duplicate for their nitrite, RxNO, and RSNO concentrations using an ozone-based reductive chemiluminescence method previously detailed [38,39]. Tissue samples were homogenized in a glass macerator with PBS, pH 7.4, with NEM/DTPA at 1:3 (weight/volume) [12]. For nitrite quantification, a sample volume of 50 µL was injected into a water-jacketed reaction chamber containing acidified tri-iodide solution and purged with nitrogen connected to an NO analyzer (Sievers Model 280 NO analyzer, Boulder, CO, USA). To measure RxNO, we used 200 µL of plasma or 300 µL of the tissue homogenate, which were treated with acid sulfanilamide (10% sulfanilamide in 1 M/L HCl) for 5 min prior to injection into the acidified tri-iodide solution. To distinguish RSNO from other NO-related species, subtractive measurements were carried out using corresponding sample aliquots treated with HgCl_2_ (2%) for 5 min before treatment with acid sulfanilamide for 3 min prior to injection into acidified tri-iodide solution. The concentrations of nitrite, RxNO, and RSNO in the tissues were normalized based on the wet weight of tissue or volume of plasma [17].

### 2.6. Measurement of Nitrate Concentrations

Nitrate concentrations were determined in tissue homogenates and plasma aliquots in duplicate using an ozone-based reductive chemiluminescence assay, as previously detailed [17]. Nitrate was measured by injecting 20 µL of plasma or 50 µL of tissue homogenate into a solution containing vanadium (III) chloride (VCl_3_) in 1 M hydrochloric acid at 95 °C [40]. The concentrations of nitrate in tissues were normalized based on the weight of macerated tissue, whereas plasma concentrations were normalized by the volume of plasma.

### 2.7. Measurement of Non-Protein Thiol Concentrations

Non-protein thiol (NPT) concentrations were measured in the plasma, brain, heart, and liver, as previously described [28,41] with minor modifications. A 50 µL aliquot of the homogenate was mixed with 200 µL of deionized water, 50 µL of 50% trichloroacetic acid, and 500 µL of 0.02 M EDTA. This solution was stirred in a vortex. After 15 min it was incubated at room temperature and shaken again, and then the sample was centrifuged for 5 min at 3200× *g* at 4 °C to remove the precipitated protein. Two hundred and fifty µL of the supernatant was transferred to a new microtube and mixed with an equal volume of TRIS buffer (0.4 M, pH 8.9), along with 25 µL of 0.01 M dithionitrobenzoic acid (DTNB) in methanol. The solution was vortexed, and after 5 min, the absorbance was read at 412 nm on a BioTek Epoch Microplate reader (software version 2.06). The concentration was calculated using a standard curve of GSH in EDTA (0.02 M). Blank controls were composed of EDTA, TRIS, and DTNB [42].

### 2.8. Assessment of Protein Nitrosation by Resin-Assisted Capture (SNORAC) Method

Total nitrosated proteins were quantified using the SNO-RAC method, as previously described [43]. Proteins were extracted from liver tissue with a buffer HEN: 100 mM HEPES, 1 mM EDTA, and 0.1 mM neocuproine supplemented with protease inhibitor cocktail (Sigmafast™ Sigma; St. Louis, MO, USA) and centrifuged at 12,000× *g* at 4 °C for 10 min. The supernatant was added to 1.6 mL of blocking buffer (HEN 2x Buffer: 200 mM HEPES, 2 mM EDTA, and 0.2 mM neocuproine, pH 8.1, plus 3% SDS and 20 mM methylmethanethiosulfonate) for 20 min at 50 °C and mixed every 5 min. Then, 6 mL of pre-chilled acetone was added, and the mixture was incubated for 20 min at −20 °C to precipitate the proteins. The samples were centrifuged at 2000× *g* for 10 min at 4 °C. The pellets were washed four times with 70% acetone at room temperature and suspended in 0.5 mL of HEN buffer with 1% SDS. Subsequently, 50 µL of the sample was collected for the protein input assay and stored at −70 °C. Then, the samples were incubated overnight at 4 °C with 20 mM of ascorbate and 40 μL of thiopropyl-sepharose 6B under agitation. All steps were carried out in the absence of light. The resin was washed four times with 1 mL of HEN buffer plus 1% SDS and five times with HEN buffer diluted 1:10 with 1% SDS (HEN Buffer/10 SDS), followed by elution with HEN Buffer/10 SDS plus 2% of 2-mercaptoethanol for 1 h at room temperature. The samples were centrifuged at 2000× *g* for 5 min at 4 °C, the supernatant was collected and stored at −70° C for analysis of assay output. To quantify the percentage of total protein nitrosylation, an SDS/PAGE was performed on a 4% polyacrylamide gel, pH 6.8, at 100 V for 60 min, so that all proteins converged into a single band, favoring quantification. The gels were stained with Coomassie Blue R-250 0.05%, and nitrosated proteins were quantified from the photographic documentation (Amersham Image 600, GE Healthcare, Little Chalfont, Buckinghamshire, UK) using the ImageJ Program 1.53k (NIH, Bethesda, MD, USA). Given the low protein concentrations in the output samples, we loaded them with a protein concentration three times higher compared to that of the input samples. The percentage of protein S-nitrosation was calculated as 100% × 3(i)/(o) [44].

### 2.9. mRNA Extraction and Real-Time Quantitative Polymerase Chain Reaction (RT-qPCR)

Total RNA was extracted from previously frozen liver tissue using TRIzol reagent (Invitrogen, Waltham, MA, USA), according to the manufacturer’s instructions, and cDNA was synthesized from 1.5 µg of RNA using the High-Capacity cDNA Reverse Transcription Kit (Applied Biosystems, Waltham, MA, USA). Quantitative PCR was performed on a StepOnePlus Real-Time PCR System (Applied Biosystems, Waltham, MA, USA) with ReadyMix JumpStart™ Taq SYBR^®^ Green (Sigma-Aldrich, St. Louis, MO, USA). The primers used for RT-qPCR are listed in Table 1. The genes analyzed included the following: Mgst1, encoding microsomal glutathione S-transferase 1, which protects cells from oxidative stress and lipid peroxidation through both glutathione S-transferase (GST) and glutathione peroxidase (GPX) activities; Hmox1, encoding heme oxygenase 1, an enzyme whose reaction products exert antioxidant, anti-inflammatory, and cytoprotective effects; Keap1, a central sensor of oxidative and electrophilic stress that regulates Nfe2l2 via proteasomal degradation; and Nfe2l2 (Nrf2), a transcription factor that coordinates the cellular antioxidant response. Functional descriptions of these genes were obtained from the UniProt Knowledgebase [45]. All reactions were run in triplicate under the following cycling conditions: 95 °C for 10 min followed by 40 cycles of 95 °C for 15 s and 60 °C for 30 s. Relative gene expression was calculated using the NRQ (Normalized Relative Quantification) method, with Actb as the endogenous control [46].

### 2.10. Assessment of Oxidative Stress and Antioxidant Parameters

In order to assess antioxidant effects exerted by nitrite [8,47] and pro-oxidant effects caused by omeprazole treatment [32,33,34], a number of additional measurements were carried out as follows.

A lucigenin-derived chemiluminescence assay was used to measure the production of superoxide (O_2−_) derived from NADPH oxidase [48]. Liver samples were homogenized in 400 μL of assay buffer (pH 7.4) with the following composition: 50 mmol/L of KH_2_PO_4_, 1 mmol/L of EGTA, and 150 mmol/L of sucrose. Fifty microliters of the homogenate were transferred to a white 96-well microplate containing 5 μmol/L of lucigenin (bis-N-methyl acridinium nitrate; Sigma-Aldrich) and assay buffer. The basal luminescence was measured (30 cycles) using an Orion II luminometer (Berthold Detection Systems, Bad Wildbad, Germany). After the initial reading, the substrate NADPH (0.1 mmol/L; Sigma Aldrich) was added to the suspension (250 μL of final volume) containing the sample and lucigenin. A second reading was conducted in the luminometer. Superoxide production was calculated by subtracting the luminescence values from the second reading (after the addition of NADPH) from those obtained in the first reading (basal luminescence). Superoxide production was expressed as relative light units (RLUs) per microgram of protein, with results expressed as RLU/mg protein [49].

Hydrogen peroxide (H_2_O_2_) concentrations were determined in liver samples from treated rats using the Amplex Red assay (Thermo Fisher Scientific, Waltham, MA, USA). Briefly, liver samples were crushed, and protein extraction was performed on 50 mmol/L sodium phosphate buffer (pH 7.4), for 1 h at 4 °C. Samples were centrifuged at 10.000× *g* for 10 min, and the supernatant extract was kept on ice until protein measurement by the Bradford assay. Samples were processed as per manufacturer instructions for the Amplex™ Red H_2_O_2_ Peroxidase Assay Kit (Thermo Fisher Scientific, A22188), in the presence of horseradish peroxidase (HRP; 10 U/mL). Samples were analyzed in duplicate using a fluorescence plate reader set to an excitation wavelength of 535 nm and an emission wavelength of 590 nm, following incubation at 37 °C for 30 min. Sodium phosphate buffer was used as blank. A H_2_O_2_ solution (0 to 10 μmol/L) was used to construct a standard curve. Results were expressed as µmol/mg protein [50].

Total hydroperoxide concentrations in the liver were measured using the Ferric–Xylenol Orange (FOX) assay, which detects (inorganic and organic) peroxides through the oxidation of ferrous iron to ferric iron, forming a colored ferric–xylenol orange complex. The FOX reagent was prepared with 100 µmol/L xylenol orange, 4 mmol/L butylated hydroxytoluene, 25 mmol/L sulfuric acid, and 250 µmol/L ammonium ferrous sulfate in a 9:1 (*v*/*v*) methanol solution. Liver homogenates (200 µL) were mixed with 2 mL of FOX reagent, vortexed, incubated for 30 min at room temperature, and centrifuged at 2000× *g* for 10 min. The absorbance of the supernatant was measured at 560 nm, and the peroxide content was determined by comparing test and blank absorbance values, using hydrogen peroxide as the standard [51].

The total antioxidant capacity of hepatic tissue was determined using the DPPH (2,2-diphenyl-1-picrylhydrazyl) radical scavenging assay. In a dark environment, 0.1 mL of each sample was added to test tubes containing 3.9 mL of a freshly prepared solution of the stable free radical DPPH in methanol and thoroughly mixed using a vortex stirrer. Methanol was used as a blank. Absorbance measurements were taken at 515 nm using a UV-Vis spectrophotometer, with readings recorded every minute until the absorbance stabilized, indicating the reduction of the DPPH radical. The antioxidant capacity of the samples was quantified by comparison to a standard curve prepared with Trolox, a water-soluble vitamin E analog.

Xanthine oxidoreductase (XOR) activity was measured in the liver with a commercially available kit (Amplex^®^ Red Xanthine/Xanthine Oxidase Assay (Thermo Fisher Scientific). In this assay, XOR causes the oxidation of hypoxanthine or xanthine to uric acid and superoxide anion. This radical degrades spontaneously to hydrogen peroxide (H_2_O_2_), which, in the presence of horseradish peroxide, reacts with the Amplex^®^ Red reagent and produces the red fluorescent oxidation product resorufin [52]. The experiments were performed according to the manufacturer’ instructions. First, samples of liver tissue were homogenized in the assay kit buffer under cooling. XOR activity was measured in duplicate by adding 50 µL of the samples to wells of a microplate and incubated for 30 min at 37 °C (protected from light) with 50 µL of a working solution containing 100 µM Amplex Red reagent, 0.4 U/mL horseradish peroxidase, and 200 µM xanthine. Fluorescence was measured using in a microplate reader (Biotek Synergy H1; Promega Corp, Winooski, VT, USA; excitation wavelength of 530 nm, detection wavelength of 590 nm). The standard curve of the assay was used to calculate XOR activity, and the values were normalized based on protein content [33].

The activity of glutathione peroxidase (GPx) was determined in the liver as previously described [53]. Briefly, the liver was homogenized in PBS (400 µL, pH 7.4), and the homogenates were centrifuged at 10,000× *g* for 10 min (4 °C). Twenty µL of the supernatant or glutathione peroxidase was incubated with reduced glutathione (6 mmol/L) and NADPH (1 mmol/L) for 5 min at room temperature in PBS (pH 7.4). After incubation, 20 µL of tert-butylhydroperoxide (70 mmol/L) was added to the tubes to start the reaction. Absorbance was monitored at 340 nm throughout the experiments [53].

To determine the total superoxide dismutase (SOD) activity, the liver was homogenized in PBS (400 μL; pH 7.4) using a Potter–Elvehjem glass-to-glass homogenizer(Bellco Glass, Inc., Vineland, NJ, USA). The homogenates were centrifuged for 10 min at 10.000× *g* (4 °C). Then, the supernatants (20 µL) were transferred to a 96-well microplate. SOD activity was determined at 450 nm following the instructions of a commercially available kit (Cat. No. 19160, Sigma-Aldrich). The results were expressed as percentage (%) inhibition per mg protein.

The activity of catalase was determined in the liver. With this purpose, the liver was homogenized in 200 μL of PBS (pH 7.4). The homogenates were centrifuged (10.000 g for 10 min at 4 °C). Then, the supernatants (10 μL) were mixed with 1640 µL of PBS and 350 µL to 500 µL of H_2_O_2_ (100 mmol/L). The decomposition rate of H_2_O_2_ was monitored for 1 min at 250 nm and expressed as units (U)/mg of protein. Catalase U was defined as the amount of catalase required to decompose 1 μmol of H_2_O_2_/min [54].

### 2.11. Assessment of Biochemical Markers of Liver Toxicity

In order to detect any overt toxic effects on liver function by the nitrite/omeprazole treatment, commercially available kits (Labtest Diagnóstica, Lagoa Santa, Brazil) were used to determine the activities of lactate dehydrogenase (LDH), alanine aminotransferase (ALT), and aspartate aminotransferase (AST) in plasma and liver homogenates [55]. Assays were performed following the manufacturer’s protocols. LDH activity was determined by measuring the enzymatic conversion of lactate to pyruvate coupled to the oxidation of NADH to NAD^+^ [56]. The decrease in absorbance at 340 nm, corresponding to NADH oxidation, is directly proportional to LDH activity in the sample. ALT and AST activities were measured via a coupled enzymatic reaction, monitored by spectrophotometric detection at 540 nm. This method involves a four-step reaction sequence, allowing for accurate quantification of transaminase activity. All measurements were conducted in triplicate, and enzyme activities were expressed in U/mL or U/mg.

### 2.12. Statistical Analysis

The results are expressed as mean ± S.E.M and were analyzed using three-way ANOVA with the following factors: tissue (FT), omeprazole treatment (FO), nitrite treatment (FN), the interaction of tissue x omeprazole (FTxO), interaction of tissue x nitrite (FTxN), interaction of omeprazole x nitrite (FOxN), and interaction of tissue x omeprazole x nitrite (FTxOxN). For analysis of the results obtained with liver or plasma samples, we used two-way ANOVA with the following factors: omeprazole treatment (FO), nitrite treatment (FN), and interaction (FI), followed by a Bonferroni post hoc test. The statistical significance level was set at 95% (*p* < 0.05). All statistical analyses were performed using GraphPad Prism 8 software.

## 3. Results

### 3.1. Changes in Plasma and Tissue Concentrations of NO Metabolites Induced by Oral Nitrite Treatment, and the Effects of Omeprazole

**Figure 1:**
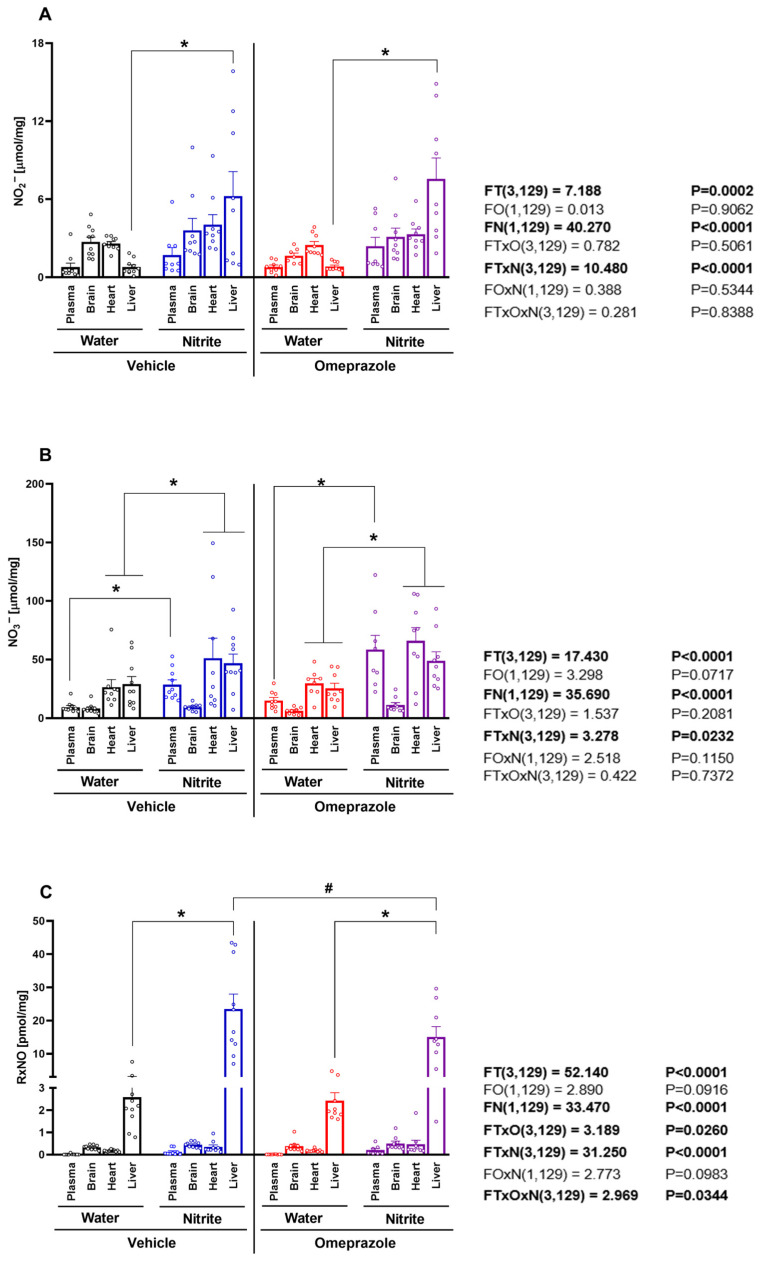
**Concentrations of NO metabolites in plasma and tissue after 14 days of treatment with sodium nitrite and/or omeprazole.** Panel (**A**) shows the nitrite concentrations in plasma, brain, heart, and liver in water- and nitrite-treated groups in the absence and presence of vehicle or omeprazole co-treatment. Panel (**B**) shows the nitrate concentrations, and panel (**C**) the total nitrosylated species (RxNO) concentrations in the same compartments and (co-)treatment groups. Data presented as mean ± S.E.M. (n = 8–10/group). The *F* values correspond to the F-statistics for three-way ANOVA (FT, tissue factor; FO, omeprazole factor; FN, nitrite factor). * *p* < 0.05 versus respective water-treated group. # *p* < 0.05 versus respective vehicle-treated group.

The concentrations of NO metabolites were measured in the plasma, brain, heart, and liver after 14 days of oral nitrite treatment. While steady-state concentrations of nitrite were distinct between compartments at baseline, nitrite treatment did not lead to a universal increase in blood and/or tissue concentrations but instead resulted in a selective increase only in the liver. Nitrite levels rose 1.2-fold in plasma and almost 8-fold in the liver (Figure 1A; *p* < 0.05), whereas only minor increases (<0.4-fold) were found in the other tissues examined. By comparison, nitrate concentrations also increased 1.5-fold in plasma and approximately 0.7-fold in the heart and liver (Figure 1B; *p* < 0.05), but no alterations were seen in the brain. Treatment with omeprazole had little effect on nitrite-induced changes in either nitrite or nitrate concentrations (Figure 1A,B; all *p >* 0.05).

The liver displayed higher baseline RxNO concentrations than plasma and the other organs examined. Treatment with nitrite increased liver RxNO concentrations approximately eight-fold (Figure 1C; *p* < 0.05), whereas no significant changes were found in other organs. Treatment with omeprazole attenuated nitrite-induced increases in RxNO concentrations to <5-fold (Figure 1C; *p* < 0.05).

Together, these results demonstrate that the liver is a major target for accumulation of bioactive NO metabolites, especially nitrite and RxNO, upon oral nitrite treatment and administration of omeprazole attenuates hepatic RxNO accumulation.

### 3.2. Nitrite Treatment Increased NPT Concentrations in the Liver, but Not in Other Tissues

**Figure 2:**
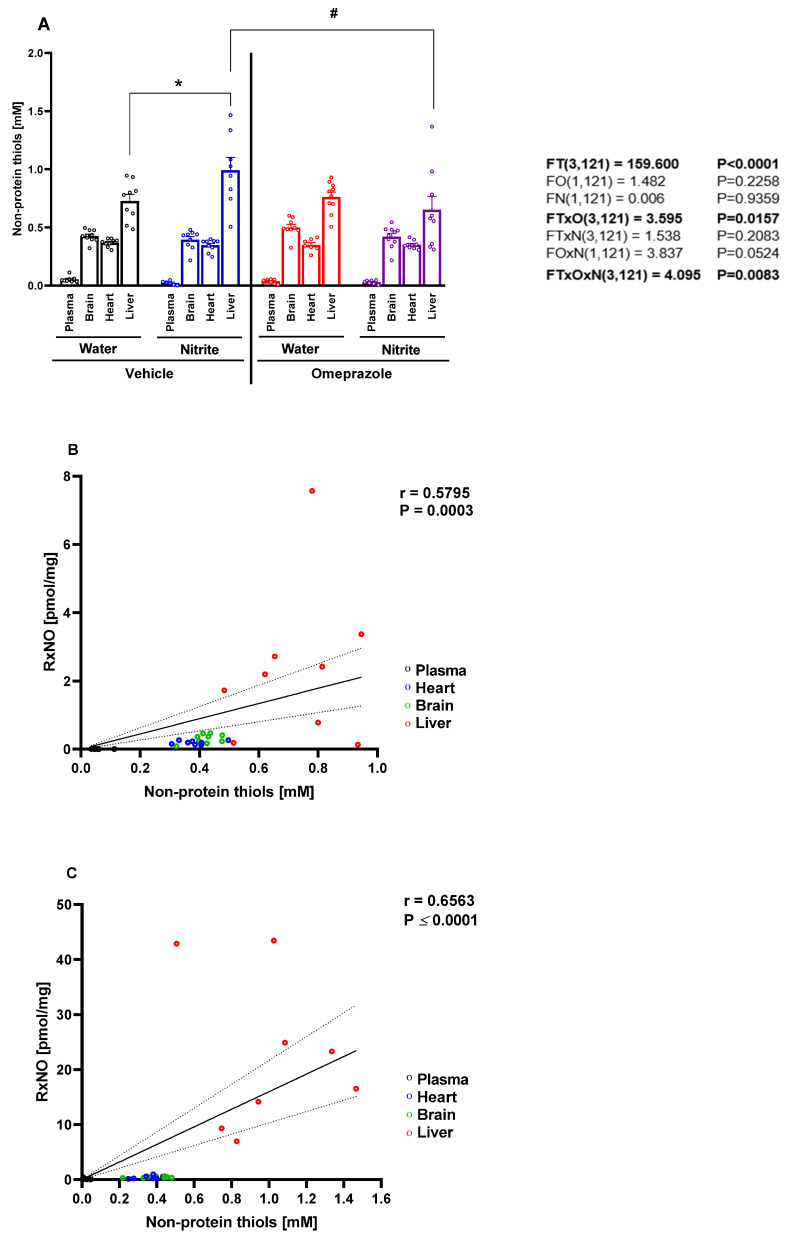
**Concentrations of non-protein thiols (NPT) in plasma, organs, saliva, and gastric juice after 14 days of treatment with sodium nitrite and omeprazole.** Panel (**A**) shows the NPT concentrations in the plasma, brain, heart, and liver in water- and nitrite-treated groups after vehicle or omeprazole pretreatment. Panels (**B**,**C**) show NPT concentrations versus RxNO concentrations in tissues from animals treated with water and with nitrite, respectively. Data presented as mean ± S.E.M. (n = 8–10/group). The *F* values correspond to the F-statistics for three-way ANOVA (FT, tissue factor; FO, omeprazole factor; FN, nitrite factor). * *p <* 0.05 versus respective water-treated group. # *p* < 0.05 versus respective vehicle-treated group. r = Pearson’s correlation coefficient. The regression line and the 95% confidence interval are plotted.

Given the remarkable accumulation of RxNO in the liver following oral nitrite treatment and the significant attenuation of this effect by co-administration of omeprazole, we sought to examine whether these changes in RxNO concentrations might be related to differences in tissue thiol concentrations. Indeed, each tissue showed distinct thiol concentrations levels that appeared to be directly related to RxNO concentrations at baseline (Figure 2A; *p* < 0.05). Supporting this idea, we found significant correlation between NPT and RxNO concentrations at baseline conditions (Figure 2B; *p* < 0.05). Oral treatment of animals with nitrite increased thiol levels by 36% only in the liver (Figure 2A; *p* < 0.05), but not significantly in the plasma, heart, or brain. Again, we found significant correlation between NPT and RxNO concentrations after nitrite treatment (Figure 2C; *p* < 0.05). Changes in the hepatic thiol concentrations upon nitrite treatment were blunted by co-administration of omeprazole (Figure 2A; *p* < 0.05).

### 3.3. Omeprazole Treatment Increases Gastric pH and Prevents Nitrite-Induced RSNO Formation and S-Nitrosylation of Hepatic Proteins

**Figure 3:**
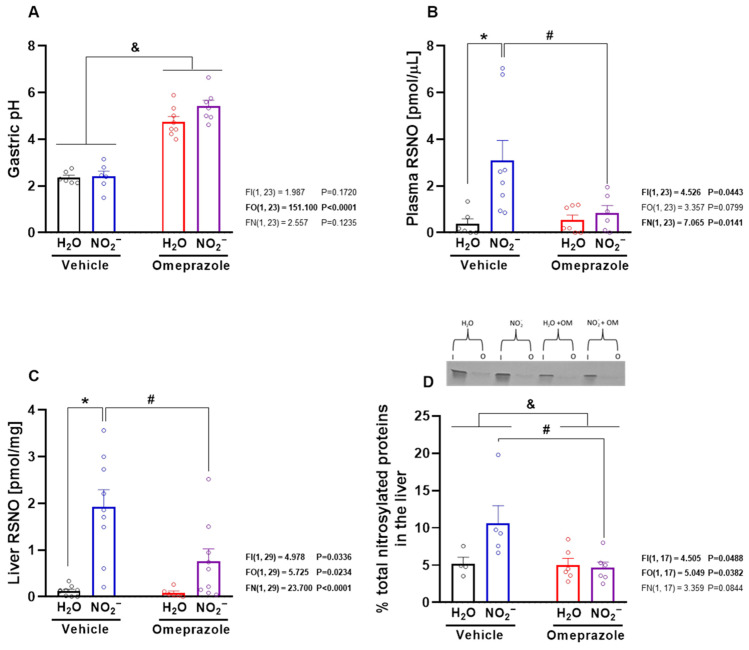
**Gastric pH, S-nitrosothiol (RSNO) concentrations in plasma and liver, and protein nitrosylation in the liver after 14 days of treatment with sodium nitrite and/or omeprazole.** Panel (**A**) shows effects of experimental treatment on gastric pH. Panels (**B**,**C**) show the RSNO concentrations in plasma and in water- and nitrite-treated groups after vehicle or omeprazole co-treatment. Panel (**D**) shows the percentage of nitrosylated proteins in the study groups with a representative SDS/PAGE gel stained with Coomassie Blue using the SNO-RAC method (“I” corresponds to input (total protein); “O” corresponds to output (nitrosylated protein)). Data presented as mean ± S.E.M. (n = 4–10/group). The *F* values correspond to the F-statistics for two-way ANOVA (FI, interaction between factors; FO, omeprazole factor; FN, nitrite factor). * *p* < 0.05 versus respective water-treated group. # *p* < 0.05 versus respective vehicle-treated group. & *p* < 0.05 for omeprazole- versus vehicle-treated groups.

The measurement of gastric pH confirmed the expected effect of omeprazole, with significant increases in gastric pH in omeprazole-treated rats (Figure 3A; *p* < 0.05). Oral nitrite treatment markedly increased RSNO concentrations in both plasma (Figure 3B; *p* < 0.05) and liver (Figure 3C; *p* < 0.05), leading to enhanced nitrosylation of hepatic proteins, as detected by the SNO-RAC assay (Figure 3D; *p* < 0.05). Interestingly, omeprazole treatment attenuated all of these nitrite-induced effects (Figure 3B–D; all *p* < 0.05).

### 3.4. Effects of Sodium Nitrite and Omeprazole Treatments on Liver mRNA Expression of Antioxidant Defense-Related Genes

**Figure 4:**
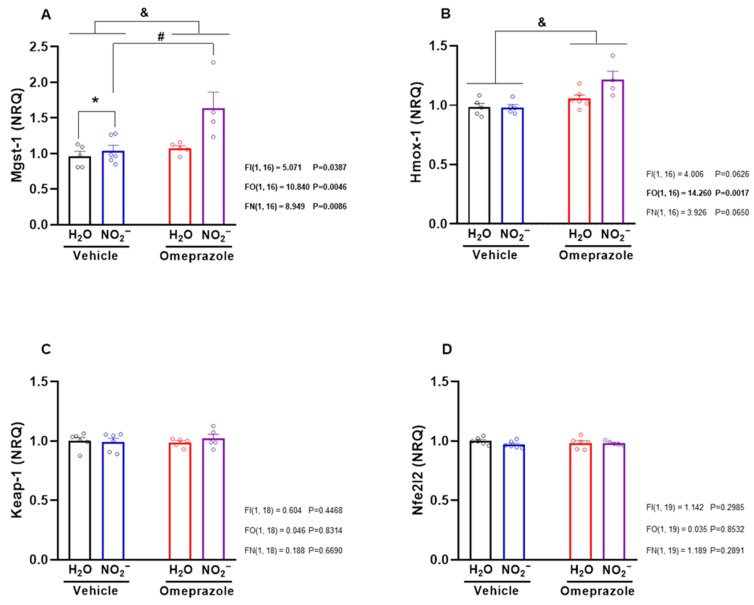
**Effects of 14-day treatment with sodium nitrite and omeprazole on liver mRNA expression of antioxidant defense-related genes.** Panels (**A**–**D**) show the relative mRNA expression of Mgst-1, Hmox-1, Keap-1, and Nfe2l2 genes, encoding for glutathione-S-transferase, heme oxygenase, Keap-1, and Nrf2, respectively, in water- and nitrite-treated groups in the presence and absence of vehicle or omeprazole co-treatment. Data presented as mean ± S.E.M. (n = 5–6/group). The *F* values correspond to the F-statistics for two-way ANOVA (FI, interaction between factors; FO, omeprazole factor; FN, nitrite factor). * *p* < 0.05 versus respective water-treated group. # *p* < 0.05 versus respective vehicle-treated group. & *p* < 0.05 for omeprazole- versus vehicle-treated groups.

Given that nitrite is known to exert pharmacological effects that may be mediated by the upregulation of antioxidant pathways, we studied the mRNA expression of key enzymes involved in such responses, specifically Mgst1 (microsomal glutathione S-transferase 1), Hmox1 (heme oxygenase 1), Keap1 (Kelch-like ECH-associated protein 1), and Nfe2l2 (nuclear factor erythroid 2–related factor 2). Nitrite treatment upregulated Mgst1 gene expression, especially in rats co-treated with omeprazole (Figure 4A; *p* < 0.05). Unexpectedly, neither nitrite nor omeprazole—alone or in combination—affected Keap1 or Nfe2l2 gene expression (Figure 4C,D; *p* > 0.05). In contrast, omeprazole upregulated Hmox1 gene expression (Figure 4B; *p* < 0.05), while nitrite alone had no effects.

### 3.5. Effects of Sodium Nitrite and Omeprazole Treatments on Liver Markers of Oxidative Stress

**Figure 5:**
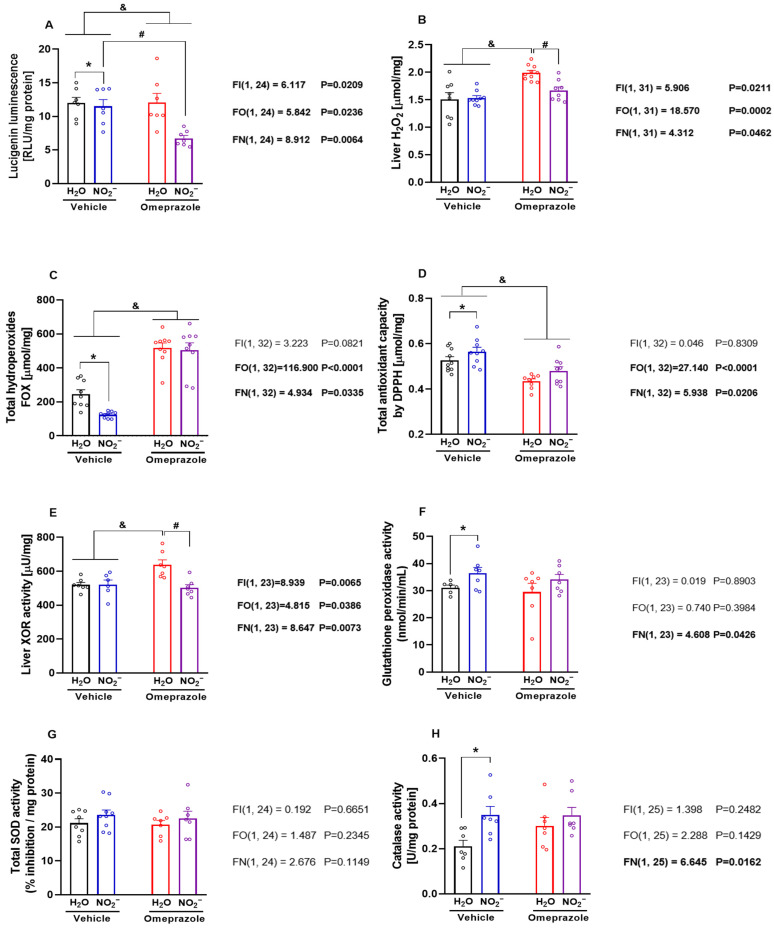
**Effects of 14-day treatment with sodium nitrite and omeprazole on liver markers of oxidative stress and redox regulating enzymes.** Panels (**A**–**D**) show NADPH-dependent superoxide production measured by lucigenin chemiluminescence, H_2_O_2_ assessed by Amplex Red assay, total hydroperoxides by the Ferric–Xylenol Orange (FOX) assay, and antioxidant capacity assessed by 2,2-diphenyl-1-picrylhydrazyl (DPPH) radical-based assay, in liver samples from rats in water- and nitrite-treated groups in the presence and absence of vehicle or omeprazole co-treatment. Panels (**E**–**H**) show, respectively, the xanthine oxidoreductase (XOR), glutathione peroxidase (GPx), superoxide dismutase (SOD), and catalase activity assessed in liver samples from rats in water- and nitrite-treated groups with and without vehicle or omeprazole co-treatment. Data presented as mean ± S.E.M. (n = 6–9/group). The *F* values correspond to the F-statistics for two-way ANOVA (FI, interaction between factors; FO, omeprazole factor; FN, nitrite factor). * *p* < 0.05 versus respective water-treated group. # *p* < 0.05 versus respective vehicle-treated group. & *p* < 0.05 for omeprazole versus vehicle-treated groups.

To explore whether or not these changes in protein expression might translate into alterations in the ability of the liver to deal with excess reactive oxygen species production, we carried out further experiments designed to examine how nitrite and omeprazole might change the redox state in hepatic tissue. Nitrite treatment resulted in the inhibition of superoxide production as assessed by the lucigenin-derived chemiluminescence assay, particularly in omeprazole-treated animals (Figure 5A; *p* < 0.05). In line with these findings, the Amplex Red assay showed that omeprazole increased liver H_2_O_2_ concentrations, and treatment with nitrite completely blunted this effect (Figure 5B; *p* < 0.05). While tissue hydroperoxide concentrations were reduced by nitrite alone, their baseline levels were robustly elevated by omeprazole (Figure 5C; *p* < 0.05), and nitrite was unable to prevent this increase. Finally, omeprazole decreased liver total antioxidant capacity, and nitrite attenuated this effect (Figure 5D; *p* < 0.05). Taken together, these results indicate that omeprazole induces pro-oxidant effects that may be attenuated by oral nitrite treatment.

Next, we examined the activity of important enzymes involved in the regulation of the tissue redox state. We found that omeprazole treatment increased XOR activity, and concomitant treatment with nitrite blunted this effect (Figure 5E; *p* < 0.05). While nitrite treatment increased GPx activity (Figure 5F; *p* < 0.05), omeprazole had no effects on this. Finally, while we found no effects of either treatment on SOD activity (Figure 5G; *p* < 0.05), treatment with nitrite increased tissue catalase activity (Figure 5H; *p* < 0.05), with no significant interaction with omeprazole.

### 3.6. Effects of Sodium Nitrite and Omeprazole Treatments on Liver Markers of Toxicity

**Figure 6:**
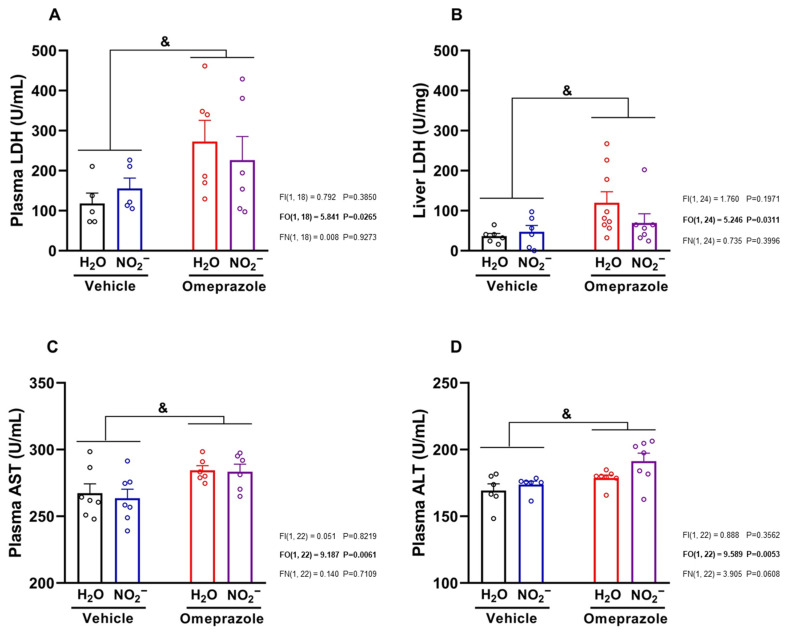
**Effects of 14 days of treatment with sodium nitrite and omeprazole on markers of hepatotoxicity.** Panels (**A**,**B**) show, respectively, plasma and liver lactate dehydrogenase (LDH) concentrations assessed in water- and nitrite-treated groups with and without vehicle or omeprazole co-treatment. Panels (**C**,**D**) show, respectively, plasma aspartate aminotransferase (AST) and alanine aminotransferase (ALT) concentrations in water- and nitrite-treated groups in the absence and presence of vehicle or omeprazole co-treatment. Data presented as mean ± S.E.M. (n = 6–7/group). The *F* values correspond to the F-statistics for two-way ANOVA (FI, interaction between factors; FO, omeprazole factor; FN, nitrite factor). & *p* < 0.05 for omeprazole- versus vehicle-treated groups.

Finally, we studied classical markers of liver toxicity and found that omeprazole treatment increased LDH both in the circulation and in the liver itself, indicative of enzyme induction (Figure 6A,B, respectively; both *p* < 0.05). Omeprazol also increased plasma AST and ALT levels (Figure 6C,D, respectively; both *p* < 0.05). Treatment with nitrite was not able to attenuate those increases in markers of liver toxicity.

## 4. Discussion

This is the first study to show that a relatively long treatment with oral nitrite results in the accumulation of NO metabolites in the liver, with a notable increase in nitrite and RxNO concentrations. The pattern of changes elicited by nitrite in the liver differed from that in other tissues and was attenuated by treatment with omeprazole. Hepatic accumulation of NO metabolites was associated with significant increases in NPT concentrations in the liver but not in other tissues. These biochemical alterations were linked with changes in gene expression of key enzymes involved in antioxidant defense and the ability of liver tissue to process and protect against oxidants. Moreover, the profile of interactions between nitrite and omeprazole in vivo is consistent with antioxidant effects of nitrite that counteract the pro-oxidant responses to omeprazole, which may result in a variety of deleterious effects [57,58,59]. The lack of response of other organs to nitrite in elevating thiol levels suggest that the liver is particularly susceptible to the effects of oral nitrite, resulting in an upregulation of overall thiol-dependent antioxidant capacity [60].

Our findings using a longer exposure to nitrite expand and confirm previous results showing the accumulation of nitrite and RxNO in the liver under more acute conditions (1 to 7 days of treatment) [18]. However, the present study shows that the liver accumulation of nitrite and RxNO promotes important alterations in the redox conditions of the liver, with several pharmacological and pathophysiological implications. The much higher concentrations of nitroso species at baseline suggests that this organ may be particularly efficient in facilitating NO storage by forming RxNO. More importantly, oral nitrite treatment caused huge increases in the concentrations of both nitrite and RxNO only in the liver, thus indicating that this organ has a marked capacity to store nitrite and its metabolites.

Given that the liver showed a very special predisposition to accumulate nitrite and RxNO (eight-fold increase), we examined the possibility that nitrite treatment-induced accumulation of liver NO metabolites could translate into antioxidant protection [8,47,61], specifically in the context of the pro-oxidant effects of omeprazole [32,33,34]. Therefore, we measured tissue thiol concentrations because thiol-based modifications of target proteins correspond to one of the most important mechanisms by which reactive oxygen species (ROS) regulate biological effects and contribute to pathophysiological mechanisms of disease conditions [60,62]. The majority of cellular thiols that react with Ellman’s regent after deproteinization will be reduced gluthatione, a major intracellular antioxidant produced in large amounts in the liver from where it is transported via circulation to other organs, possibly reflecting an antioxidant reserve capacity [63]. Interestingly, we found that nitrite treatment increased thiol concentrations only in the liver and not in plasma or other organs, strongly suggesting that nitrite improves antioxidant reserve capacity by increasing hepatic glutathione production. This effect was blunted by omeprazole co-administration, suggesting that nitrite may not fully counteract pro-oxidant alterations induced by omeprazole.

Treatment with omeprazole increased gastric pH and attenuated oral nitrite-induced increases in circulating RSNO, as previously shown [25,26]. The attenuation of oral nitrite-induced increases in both liver RSNO concentrations and total protein nitrosylation that we observed when animals were co-treated with omeprazole may be explained by the attenuation of the increases in circulating RSNO leading to attenuated transnitrosylation of liver proteins. Alternatively, approximately half of the biochemical signature measured may be attributed to S-nitrosoglutathione rather than a protein-bound S-nitrosothiol. Indeed, these results may reflect lower thiol availability for nitrosylation and impaired redox balance caused by omeprazole. Interestingly, nitrosylation of liver proteins has been reported to confer protection against acetaminophen-induced liver injury [64].

To further investigate whether the liver accumulation of nitrite and RxNO counteracts pro-oxidant responses to omeprazole, we carried out a series of studies to determine markers of liver redox conditions. Two decades ago, nitrite was shown to regulate gene expression, including heme oxygenase 1 [65]. We therefore examined the gene expression of Mgst1 (microsomal glutathione S-transferase 1) [66], Hmox1 (heme oxygenase 1) [67], Keap1 (Kelch-like ECH-associated protein 1) [68], and Nfe2l2 (nuclear factor erythroid 2–related factor 2) [69]. Nitrite treatment increased the mRNA expression of Mgst1 in rats also treated with omeprazole, and this result indicates that treatment with both nitrite and omeprazole interacts in such a way to enhance the expression of glutathione S-transferase 1. This enzyme plays a central role in cellular detoxification and protection against oxidative stress by facilitating the elimination of electrophilic compounds following conjugation with glutathione [70]. However, we found no effects on the expression of Keap1 and Nfe2l2 gene, an unexpected finding given that previous studies indicated this pathway as a contributor to the antioxidant responses to oral nitrite administration [47]. The increase in Hmox1 gene expression as a result of treatment with omeprazole, particularly when combined with nitrite, suggests a complex interaction between both treatments that result in protection associated with increased CO formation, even though nitrite has previously been shown to downregulate heme oxygenase 1 expression [65].

To further explore the interaction of nitrite and omeprazole in the context of reactive oxygen species handling and tissue redox status, we directly measured ROS in liver tissue. We found that nitrite treatment resulted in major reduction in superoxide production, particularly in omeprazole-treated animals. This finding was paralleled by nitrite blunting of omeprazole-induced increases in liver H_2_O_2_ concentrations. However, the measurements of total hydroperoxide concentrations and total antioxidant capacity indicate pro-oxidant effects of omeprazole that were not attenuated by nitrite treatment. We next explored the activity of enzymes involved in redox regulation. Interestingly, omeprazole increased XOR activity, as previously found in other studies [32,33], and nitrite blunted this effect. GPx, SOD, and catalase activity were not affected by omeprazole treatment, even though nitrite treatment increased GPx and catalase activity in control animals. While not all of our findings fit together, they are consistent with the overall conclusion that omeprazole increases ROS formation in the liver and that nitrite co-administration partly inhibits this effect, probably as a result of XOR inhibition and increased expression of Mgst1 gene. Omeprazole treatment was associated with increased concentrations of markers of hepatotoxicity, and nitrite treatment was unable to protect against this effect.

Our findings may have clinical implications, particularly for patients taking omeprazole, which is often, though controversially, regarded as a safe drug [71,72,73,74]. However, mounting evidence indicates that omeprazole and other proton pump inhibitors may cause a variety of unwanted effects and lead to clinical complications, including hypomagnesemia [75,76], renal diseases [77,78], *Clostridium difficile* infection [79], pneumonia [80,81], and osteoporotic fractures [82,83]. Regarding their toxicity, the literature mainly includes case reports, with very few studies suggesting hepatotoxicity [84,85,86,87]. However, a recent study has indicated an association between the use of proton pump inhibitors and cholestasis or cholestatic hepatitis [84], which aligns with other studies showing abnormal hepatic function as a common adverse effect [88]. It is not known whether treatment with oral nitrite or dietary intake of nitrate-rich food/supplementation with nitrate (which results in an increased supply of the body with nitrite) could help prevent such complications, and adverse effects remain to be determined. Supporting this suggestion, nitrate treatment has shown beneficial effects in non-alcoholic fatty liver disease [89,90].

## 5. Conclusions

In conclusion, our results show that oral nitrite treatment accumulates nitrite and RxNO in the liver in such a way that increased concentrations of nitrosated proteins and reduced glutathione (likely caused by an increase in glutathione production) is associated with the upregulation of Mgst1 expression, thus counteracting pro-oxidant mechanisms induced by omeprazole treatment (or other unfavorable conditions). Our results suggest new mechanisms by which oral nitrite treatment may prevent part of the hepatotoxicity elicited by omeprazole treatment.

## Figures and Tables

**Table 1:** **List of oligonucleotides used for RT-qPCR**.

Gene	Sequence	NCBI Accession #	Source
Mgst1 (Forward)	5′-CCGTCACCCTCTGATTGATTTA-3′	P08011	Designed by the authors
Mgst1 (Reverse)	5′-TCCTGATTTCTCTGCTCCTTTC-3′		Designed by the authors
Hmox1(Forward)	5′-CCTGTGTCTTCCTTTGTCTCTC-3′	P06762	Designed by the authors
Hmox1 (Reverse)	5′-GGGCTCTGTTGCAGGATTT-3′		Designed by the authors
Keap-1 (Forward)	5′–TCCTCCAGCCCAGTCTTTA-3′	P57790	Designed by the authors
Keap-1 (Reverse)	5′-CCGTGTAGGCGAACTCAATTA-3′		Designed by the authors
Nfe2l2 (Forward)	5′-AATTGCCACCGCCAGGACTA-3′	O54968	Designed by the authors
Nfe2l2 (Reverse)	5′-CAAACACTTCTCGACTTACCCC-3′		Designed by the authors
Actb (Forward)	5′-CTAAGGCCAACCGTGAAAAG-3′	P60711	[46]
Actb (Reverse)	5′-AACACAGCCTGGATGGCTAC-3′		[46]

# corresponds to “number”.

## Data Availability

Data are contained within the article.
